# Global Incidence and Mortality Trends due to Adverse Effects of Medical Treatment, 1990–2017: A Systematic Analysis from the Global Burden of Diseases, Injuries and Risk Factors Study

**DOI:** 10.7759/cureus.7265

**Published:** 2020-03-14

**Authors:** Javaid Nauman, Elpidoforos S Soteriades, Muhammad Jawad Hashim, Romona Govender, Reem Saif Al Darmaki, Reem Juma Al Falasi, Shreesh Kumar Ojha, Shamaila Masood-Husain, Syed Fahad Javaid, Moien AB Khan

**Affiliations:** 1 Epidemiology and Public Health, Institute of Public Health, College of Medicine and Health Sciences, United Arab Emirates University, Al Ain, ARE; 2 Family Medicine, College of Medicine and Health Sciences, United Arab Emirates University, Al Ain, ARE; 3 Family Medicine, United Arab Emirates University, Al Ain, ARE; 4 Family Medicine, Ambulatory Health Care Services, Al Ain, ARE; 5 Pharmacology and Therapeutics, College of Medicine and Health Sciences, United Arab Emirates University, Al Ain, ARE; 6 Family Medicine, Parchmore Medical Centre, London, GBR; 7 Psychiatry and Behavioral Science, College of Medicine and Health Sciences, United Arab Emirates University, Al Ain, ARE

**Keywords:** medical errors, adverse events, patient safety, global burden, complication of treatment, health policy, health care system, global health, side effects of medical treatment, public health

## Abstract

Aim

To quantify the global incidence and mortality of adverse effects of medical treatment (AEMT) and forecast the possible emerging trends of AEMT.

Materials and methods

We analyzed the latest data from the Global Burden of Disease (GBD) 2017 study. We describe the burden of AEMT based on age- and region-specific incidence and mortality rates between 1990 and 2017. Additionally, we evaluated the change of burden due to AEMT by different periods between 1990 and 2017, and compared the age-standardized incidence and mortality rates among different World Health Organization (WHO) regions.

Results

Globally, AEMT incidence rates varied across WHO regions and countries. The estimated age-standardized average incidence rates of AEMT were 309 [95% uncertainty interval (UI), 270 to 351], 340 (298 to 384), 401 (348 to 458), and 439 (376 to 505) per 100,000 population across the world in 1990, 2000, 2010, and 2017, respectively, showing an increasing trend in the new occurrence of adverse events. The incidence rate among women (469/100,000) was higher compared to men (409/100,000) in 2017. Between 1990 and 2017, we observed an upward trend in the incidence rates of AEMT across global regions, with a substantial increase in the incidence by 42% (27 to 57) between the years 1990 and 2017, translated to an annualized rate of incline of 1.5%. In the age group of 60-64 years, the incidence rates increased by 96% in 2017 compared to 1990. The global incidence rate due to AEMT is forecasted to increase to 446.94 (433.65 to 460.22) by 2020, 478.49 (376.88 to 580.09) in 2030, and to reach 510.03 (276.58 to 743.49) per 100,000 by 2040. We observed a decline in mortality rates due to AEMT across global regions, and the annualized rate of mortality change was -0.90 percentage points between 1990 and 2017. Overall, the AEMT mortality rate was higher in men (1.73/100,000) than in women (1.48/100,000), and age-specific mortality rates showed a bimodal increase between the age group of birth to one year, and an increase in the age group of 65 years and above. The global mortality rate due to AEMT is expected to be 1.55 (1.48 to 1.61) in 2020, 1.37 (0.88 to 1.86 ) in 2030 and 1.2 deaths per 100,000 (0.08 to 2.32) by 2040.

Conclusion

Using the GBD 2017 study data, we found an increase in the incidence of AEMT, and an overall decrease in the mortality rate between 1990 and 2017, with varying estimates between different countries and regions, gender and age groups. The forecast analysis displayed the same trends - an increase in AEMT incidence and a decline in mortality between 2020 and 2040. The high burden of AEMT warrants the implementation of robust policies in the healthcare system including appropriate patient safety training for the healthcare professionals, and safe culture of feedback with the implementation of electronic medical records to achieve WHO patient safety strategy goals.

## Introduction

An adverse effect of medical treatment (AEMT) is defined as an “unintended injury due to medical care leading to an extended hospital stay, disability at the time of discharge, serious and devastating long-term irreversible consequences or death” [1,2]. AEMT is caused by medical mismanagement rather than by the underlying disease of patients and has a widespread occurrence in healthcare settings, with an estimated one in 10 patients being harmed while receiving care [2-5]. In the United States, AEMT is the third leading cause of death, while in low and middle-income countries (LMIC), one-third of deaths are attributed to AEMT [5,6]. Interestingly, four out of 10 patients are harmed in primary care settings, which results in hospitalization accounting for 6% of total hospital bed days [7]. Medication errors account for 1% of the global healthcare burden of AEMT, or 42 billion USD annually [8]. Similarly, poor quality of care resulted in more than eight million deaths in 2015, equating to six trillion USD of economic impact in LMICs [9].

Although AEMTs are a global health concern, they are preventable in 80% of cases [6]. Therefore, WHO have launched key strategies and aim to reduce the incidence of AEMT by 50% by the year 2022 [10]. However, limited data exist on the levels, trends and patterns of the causes and effects of AEMT globally and in each region as defined by WHO over a specific period. Earlier reports relating to patient safety and AEMT were primarily based on random reviews of selected medical records and hospitals during a specific time [11,12]. Therefore the present study aims to analyze global and region-specific patterns of incidence and mortality, and predict possible emerging trends due to AEMT using the GBD injuries and risk factor study for the period 1990-2017.

## Materials and methods

The Global Burden of Diseases, Injuries and Risk Factors Study 2017 (GBD 2017) is a systematic global collaborative research project to measure and quantify the degree of health loss due to disease, injury, and risk factors by age, gender and geographic regions through specific points in time [13]. The data set is managed by the Institute of Health Metrics and Evaluation at the University of Washington, Seattle [14,15].

The data entered into the GBD database come from various sources including inpatient, outpatient, and emergency department hospital records, as well as analysing hospital claims. The GBD database uses the International Classification of Diseases (ICD) codes to extract data. Injury due to adverse effects of treatment is defined using ICD-9 and ICD-10.

Morbidity and years of disability due to adverse effects are estimated using data obtained from medical treatment hospital records, emergency department records, insurance records, and surveys. The information is later pooled through various sophisticated mathematical modeling techniques and statistical estimates by age, cause, country, gender, and year. A detailed description of the methodology of the GBD study is provided elsewhere [16,17].

Data sources

We retrieved and analyzed the epidemiological data attributed to AEMT from the latest GBD 2017 dataset and quantified the results by the WHO regional groups [15]. Age-standardized rates were used to compensate for the variation in different age groups. For our study, we extracted age-standardized incidence and mortality with 95% uncertainty intervals using the GBD interactive data visualization and GBD Compare tools.

World Health Organisation regional groups

We used the GBD 2017 data based on the WHO regions (African Region, Region of the Americas, Southeast Asian Region, European Region, Eastern Mediterranean Region, and Western Pacific Region: https://www.who.int/chp/about/regions/en). The regional groups are administrative areas with the representation of different countries that meet to address issues pertaining to the geographic region. In addition, they develop technical cooperation strategies for regional health development and provide policies for health promotion, disease and injury prevention by working together with intergovernmental bodies and other non-governmental organizations (NGOs).

Adverse effect

The AEMTs are ICD coded, and a detailed list of the ICD-9 and ICD-10 codes used for AEMT in healthcare settings and hospital claim analysis is available in the supplementary paper of the GBD [18].

Uncertainty intervals

The burden of disease estimates may be uncertain due to errors in input data, statistical models, and data adjustments. Uncertainty intervals (UIs) are a range of values that are likely to include the correct estimate of health loss for a given cause. The UIs are expressed as 2.5 and 97.5 centile values [16].

Incidence (per 100,000)

Incidence in this article refers to the number of new cases or individuals who experience an AEMT event during a particular time period. Estimation of incidence is carried out by taking the number of new cases in a year, which is then divided by the mid-year population of that country[12].

Mortality rate (per 100,000)

The number of deaths caused by AEMT during medical treatment per year among the entire population defines the mortality rate. The mortality rate is expressed in deaths per 100,000 individuals per year. The GBD study defines a site-year as a contribution of data related to death in a given year by a country, state, or another geographical unit [19]. Specifically, a total number of 1,485 site-years were included for the GBD 2017 dataset [16,17]. The age-standardized mortality rate with 95% UIs by geography, gender, and year has been analyzed by the WHO.

Trends and percentage change

The change in percentage between the two values defines the trends or degrees of change. The percent change could increase, decrease or remain the same, which we calculated at different points between the years 1990 and 2017. The percentage difference between the two values was calculated to show changes in age-specific mortality and incidence rates between 1990 and 2017.

Statistical analysis

The statistical package for the social sciences (SPSS Statistics for Windows, version 23.0, Chicago, IL, USA) was used for statistical analysis. We estimated the percentage difference of incidence and mortality for every WHO region with the available GBD estimates between two time points. Various sources may contribute to the uncertainty of the estimates including data availability, model specifications, and sample size variability between data sources. We calculated the 95% UIs with the 2.5th and 97.5th percentiles based on cause-specific model estimations for each GBD location, sex, age group, and time points between 1990 and 2017 [20]. We used the SPSS time series modeler with the expert modeler option without events to predict future AEMT incidence and mortality when none of the observed values were marked as outliers.

## Results

Incidence

In Table 1 we present the estimated new cases per 100,000 with 95% UIs due to AEMT. In 2017, the estimated age-standardized rate of a new occurrence of an adverse effect that resulted in a need for medical treatment was 438.97 per 100,000 population [95% UI, (376.41 to 505.28)]. These numbers translated into 32.9 million people being affected globally per year in 2017. The age-standardized average incidence rates were 309, 340, 401, and 439 per 100,000 across the world in 1990, 2000, 2010, and 2017, respectively (Table 1), showing an increasing trend in the occurrence of adverse events globally. The incidence rates due to AEMT varied between countries and regions. For example, we observed a higher incidence of AEMT in 2017 in countries such as Australia [2232.35 (1930.15 to 2577.7)], and the United States [2629.95 (2242.94 to 3077.48)]. Conversely, countries such as Ghana [277.97 (239.81 to 315.69)], India [167.26 (140.4 to 194.8)] and Brazil [95.09 (83.8 to 107.53)] showed lower incidence rates. Within the WHO regions, the Americas had the highest incidence of 1,160.99 (991.66 to 1359.14), compared to the lowest incidence in the Southeast Asian region [155.98 (130.7 to 182.52)] per 100,000 in 2017.

**Table 1:** Global Age-standardized incidence rates per 100,000 population due to adverse effect of medical treatment by World Health Organization regions

	Incidence (95% Uncertainty Intervals)				Percentage Change (95% Uncertainty Intervals)		
	1990	2000	2010	2017	1990 to 2017	2000 to 2017	2010 to 2017
Global	309.39 (270.1 - 350.95)	339.5 (298.14 - 383.64)	401.3 (347.86 - 458.43)	438.97 (376.41 - 505.28)	41.88 (26.97 to 56.78)	29.29 (17.16 to 41.41)	9.38 (5.2 to 13.55)
African Region	169.02 (147.68 - 189.33)	166.37 (145.17 - 186.97)	172.04 (148.91 - 195.29)	180.53 (153.6 - 208.23)	6.80 (-7.18 to 20.78)	8.51 (-2.36 to 19.38)	4.93 (-1.4 to 11.26)
Angola	167.41 (144.65 - 189.35)	161.68 (140.61 - 183.03)	170.85 (146.21 - 195.77)	174.38 (146.94 - 202.30)	4.16 (-9.70 to 18.02)	7.85 (-2.97 to 18.67)	2.06 (-4.29 to 8.41)
Ghana	248.23 (220.32 - 277.28)	256.75 (228.39 - 288.02)	273.81 (239.7 - 306.16)	277.97 (239.81 - 315.69)	11.98 (-1.99 to 25.95)	8.26 (-2.81 to 19.33)	1.51 (-4.84 to 7.86)
Kenya	133.44 (114.50 - 151.92)	126.69 (108.79 - 144.37)	135.76 (114.91 - 156.79)	147.4 (123.24 - 173.12)	10.46 (-3.89 to 24.81)	16.34 (4.97 to 27.7)	8.57 (2.08 to 15.05)
South Africa	175.60 (150.95 - 199.15)	184.53 (158.47 - 209.88)	190.93 (161.98 - 219.55)	187.37 (155.96 - 220.88)	6.70 (-7.61 to 21.01)	1.53 (-9.84 to 12.9)	-1.86 (-8.35 to 4.63)
Eastern Mediterranean Region	205.33 (179.88 - 230.84)	206.34 (180.95 - 232.5)	226.21 (196.19 - 256.49)	235.91 (200.67 - 271.80)	14.89 (0.46 to 29.31)	14.33 (2.9 to 25.75)	4.28 (-2.30 to 10.86)
Egypt	201.04 (174.07 - 228.94)	200.16 (172.03 - 229.7)	218.59 (185.74 - 252.74)	239.63 (200.99 - 280.93)	19.19 (4.55 to 33.82)	19.71 (8.07 to 31.34)	9.62 (2.90 to 16.33)
Saudi Arabia	409.87 (361.35 - 460.94)	432.61 (384.92 - 487.88)	444.89 (391.59 - 506.17)	399.52 (344.03 - 455.67)	-2.52 (-17.42 to 12.38)	-7.64 (-19.51 to 4.23)	-10.19 (-17.03 to -3.34)
European Region	285.85 (247.06 - 327.41)	279.16 (244.37 - 316.69)	314.69 (276.09 - 356.97)	322.76 (277.51 - 371.46)	12.91 (-1.89 to 27.71)	15.61 (3.91 to 27.3)	2.56 (-4.22 to 9.34)
Germany	374.43 (317.78 - 433.56)	403.05 (342.97 - 467.16)	400.88 (345.95 - 466.18)	420.28 (359.45 - 489.61)	12.24 (-2.75 to 27.23)	4.27 (-7.66 to 16.2)	4.83 (-2.10 to 11.76)
Kazakhstan	241.37 (207.64 - 275.22)	205.04 (177.24 - 235.4)	258.09 (221.08 - 297.44)	282.10 (239.15 - 326.23)	16.87 (1.68 to 32.05)	37.58 (25.57 to 49.58)	9.3 (2.19 to 16.4)
Poland	252.75 (217.10 - 290.25)	236.16 (214.33 - 263.82)	352.4 (312.55 - 397.60)	394.59 (330.85 - 456.81)	56.11 (40.66 to 71.55)	67.08 (54.84 to 79.31)	11.97 (4.68 to 19.25)
Russian Federation	171.98 (147.83 - 196.75)	159.52 (137.2 - 181.81)	209.7 (179.65 - 241.73)	212.49 (179.76 - 245.79)	23.55 (7.88 to 39.21)	33.20 (21.7 to 44.69)	1.33 (-6.12 to 8.78)
Sweden	401.44 (338.40 - 468.91)	699.36 (591.05 - 810.3)	1052.27 (890.99 - 1226.73)	592.73 (496.75 - 692.31)	47.65 (31.58 to 63.71)	-15.24 (-26.99 to -3.48)	-43.67 (-51.31 to -36.02)
Ukraine	132.09 (112.47 - 153.00)	107.85 (90.46 - 125.44)	139.56 (117.21 - 163.44)	176.21 (147.5 - 206.18)	33.40 (16.92 to 49.87)	63.38 (52.23 to 74.52)	26.26 (21.67 to 30.84)
United Kingdom	297.96 (247.19 - 353.42)	187.57 (165.23 - 210.8)	199.4 (177.41 - 223.65)	215.12 (187.8 - 244.26)	-27.8 (-44.81 to -10.78)	14.68 (4.4 to 24.95)	7.88 (0.9 to 14.85)
Region of the Americas	762.05 (664.31 - 865.64)	906.02 (796.24 - 1024.28)	1102.73 (950.42 - 1271.67)	1160.99 (991.66 - 1359.14)	52.35 (34.73 to 69.96)	28.14 (16.86 to 39.41)	5.28 (-2.08 to 12.64)
Argentina	489.35 (431.97 - 550.07)	591.03 (522.32 - 665.53)	665.4 (580.67 - 755.86)	671.18 (579.62 - 770.69)	37.15 (21.69 to 52.6)	13.56 (2.96 to 24.15)	0.86 (-6.18 to 7.90)
Brazil	78.98 (69.08 - 89.32)	101.36 (91.12 - 112.10)	102.05 (91.53 - 113.06)	95.09 (83.8 - 107.53)	20.39 (4.34 to 36.43)	-6.18 (-17.1 to 4.74)	-6.82 (-14.2 to 0.56)
Cuba	119.54 (103.06 - 136.82)	114.21 (97.45 - 131.70)	132.68 (110.68 - 156.24)	159.11 (132.34 - 188.58)	33.10 (16.6 to 49.59)	39.31 (28.78 to 49.83)	19.92 (12.53 to 27.3)
Mexico	97.15 (82.94 - 111.66)	89.07 (78.67 - 100.15)	109.17 (96.72 - 122.52)	112.81 (96.19 - 129.87)	16.11 (-1.08 to 33.3)	26.65 (15.88 to 37.41)	3.33 (-4.02 to 10.68)
United States	1545.99 (1336.90 - 1755.56)	1935.51 (1689.67 - 2186.69)	2414.57 (2077.29 - 2795.06)	2629.95 (2242.94 - 3077.48)	70.11 (51.63 to 88.58)	35.87 (24 to 47.73)	8.92 (1.24 to 16.59)
Southeast Asian Region	126.46 (108.88 - 144.09)	132.67 (113.75 - 151.11)	136.87 (116.21 - 157.95)	155.98 (130.7 - 182.52)	23.34 (9.36 to 37.31)	17.56 (5.91 to 29.2)	13.96 (6.21 to 21.7)
China	310.63 (265.53 - 356.74)	329.97 (280.81 - 382.85)	419.35 (356.64 - 486.25)	507.98 (431.01 - 591.16)	63.53 (44.69 to 82.36)	53.94 (41.58 to 66.29)	21.13 (13.28 to 28.97)
India	121.99 (104.84 - 138.7)	128.68 (110.45 - 146.65)	141.02 (119.58 - 161.96)	167.26 (140.4 - 194.80)	37.10 (23.11 to 51.08)	29.98 (19.23 to 40.72)	18.60 (10.93 to 26.26)
Indonesia	131.01 (108.93 - 154.31)	141.99 (117.77 - 167.64)	110.06 (89.52 - 131.77)	107.56 (85.52 - 132.95)	-17.89 (-31.75 to -4.02)	-24.24 (-34.93 to -13.54)	-2.27 (-9.99 to 5.45)
Western Pacific Region	277.16 (237.50 - 317.81)	305.03 (261.84 - 351.60)	381.91 (327.58 - 439.95)	453.93 (386.46 - 526.81)	63.77 (49.13 to 78.4)	48.81 (37.02 to 60.59)	18.85 (14.26 to 23.43)
Australia	1071.67 (927.06 - 1223.24)	1718.18 (1503.14 - 1954.90)	2179.34 (1903.48 - 2499.87)	2232.35 (1930.15 - 2577.7)	108.30 (93.94 to 122.65)	29.92 (18.34 to 41.49)	2.43 (-5.31 to 10.17)
Fiji	275.76 (239.18 - 313.87)	286.99 (250.02 - 325.05)	303.78 (263.29 - 347.68)	334.29 (285.57 - 386.65)	21.22 (6.9 to 35.53)	16.48 (4.88 to 28.07)	10.04 (2.32 to 17.75)
Japan	115.96 (96.67 - 136.43)	122.13 (102.12 - 143.78)	111.65 (98.37 - 125.33)	113.59 (99.83 - 127.80)	-2.04 (-16.46 to 12.38)	-6.99 (-18.4 to 4.42)	1.73 (-6.13 to 9.59)

**Figure 1:**
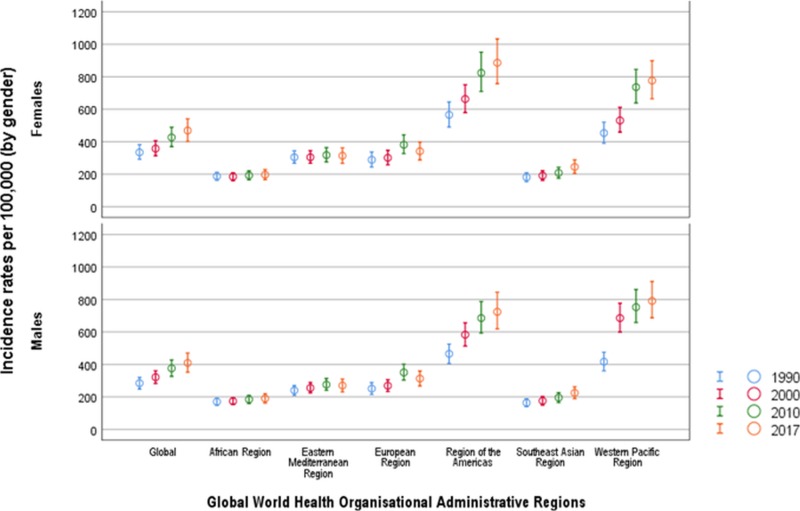
Age-standardized incidence rates due to adverse effect of medical treatment by Global World Health Organization regions between 1990 and 2017 Incidence rates are per 100,000 population. Circles represent summary estimates of incidence and error bars represent 95% uncertainty intervals.

In Figure 1 we delineate age-standardized incidence rates with 95% UIs from the six WHO regions between 1990 and 2017. In addition, the incidence rate among women (468.68/100,000) was higher compared to men (409.27/100,000) in 2017. The percentage change in AEMT incidence also varied between countries and regions. Between 1990 and 2017, we observed an upward trend in the incidence rates occurring due to AEMT across global regions, with a substantial increase in incidence of 41.88% (26.97 to 56.78) between the years 1990 and 2017, with an annualized rate of incline of 1.49% (Table 1). Although the global annualized trend is set to increase, there is a slowing-down trend between the years 2000 and 2017, showing an annualized rate of 1.63% compared to a rate of 1.17% between 2010 and 2017. Rates of change for AEMT between 1990 and 2017 vary widely between WHO regions and countries, from a 63.77% (49.13 to 78.4) increase in the Western Pacific region to an increase of 12.91% (-1.89 to 27.71) in the European region. All regions show a significant increase of AEMT from 1990 to 2017. Interestingly, between 2010 and 2017, the Western Pacific region showed the highest rate of incline of 18.85% (14.26 to 23.43) compared to the least increasing trend in the European region [2.56 (-4.22 to 9.34)].

The incidence of AEMT in 2017 showed an increasing trend compared to 1990, with a 108% increase in Australia, a 70% increase in the United States, and a 64% increase in China. In comparison, the United Kingdom showed a positive change with a 27.8% decrease in the number of new occurrences of AEMT. In addition, Japan saw a decline in AEMT incidence of 2% in 2017 compared to 1990.

Across different countries in the WHO regions between 2010 and 2017, the highest increasing trends in incidence between 2010 and 2017 were seen in Ukraine [26.26% (21.67 to 30.84)], China [21.13% (13.28 to 28.97)], Cuba [19.92% (12.53 to 27.3)], India [18.6% (10.93 to 26.26)], and Fiji islands [10.04% (2.32 to 17.75)]. In contrast, during the period 2010 to 2017, the incidence rates of AEMT were -43.67% (-51.31 to -36.02), -10.19% (-17.03 to -3.34), -2.27% (-9.99 to 5.45), and -1.86% (-8.35 to 4.63) in Sweden, Saudi Arabia, Indonesia, and South Africa, respectively.

Incidence by age and gender

**Figure 2:**
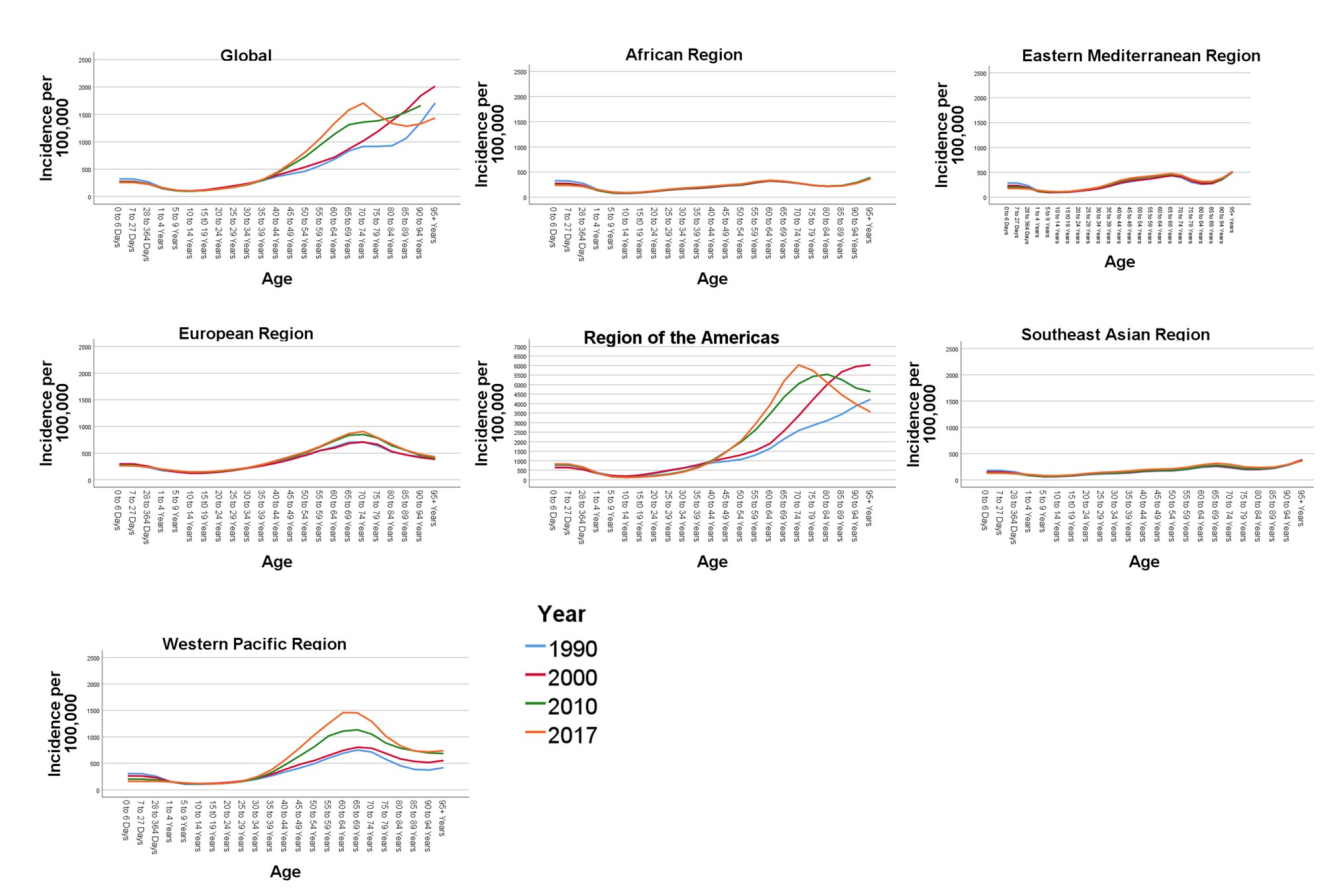
Age-specific incidence rates due to adverse effect of medical treatment by Global World Health Organisation administrative regions between 1990 and 2017 The figure panes depict the overall incidence rates per 100,000 population globally and across the six World Health Organization (WHO) regions (African region, Eastern Mediterranean region, European Region, Region of the Americas, Southeast Asian Region and the Western Pacific Region) during the year 1990, 2000, 2010 and 2017.

In Figure 2 we show the trends of age-specific incidence rates of AEMT globally by WHO regions between 1990 and 2017. There is a higher incidence rate of AEMT in the early years of life (0-364 days). All trend lines are gradually ascending from 10-14 years of age and upward. The steepest rise in incidence is observed in the age group 40-45 years and upward, with an extremely high incidence rate starting from 60-64 years. In the age group 60-64, the incidence number increased by 96.21% in 2017 compared to the year 1990. This strong trend of increased AEMT incidence continues for the 65 and over age group which reflects a higher risk of adverse events in extremes of age.

Overall, the incidence rates due to AEMT were higher in women than in men (Figure 1). In 2017, the global incidence rate was 468.68 per 100,000 in women compared to 409.27 in men per 100,000 population, reflecting a 12% higher incidence rate in women. However, between 1990 and 2017, there was a 43% increase in the incidence of AEMT among men, while there was only a 20% increase in incidence among women (Figure 1, Table 1).

Forecasted incidence trend

**Figure 3:**
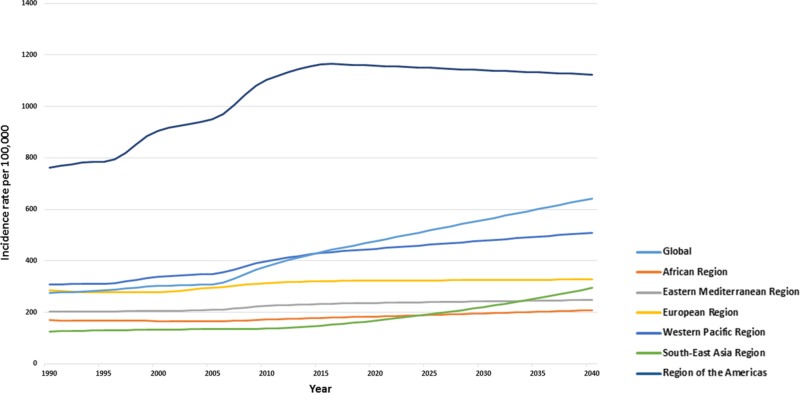
Forecasted age-standardized incidence trends across Global World Health Organization regions till 2040

The incidence for AEMT is forecasted to increase to 446.94 (433.65 to 460.22) by 2020, to 478.49 (376.88 to 580.09) by 2030, and to reach epidemic proportions of 510.03 per 100,000 (276.58 to 743.49) by 2040 (Figure 3).

The greatest increase in AEMT incidence is expected to be in the region of the Americas: 1,158.14 (1106.48 to 1209.80) in 2020, 1,141.03 (745.91 to 1,536.15 ) in 2030, 1,123.92 (216.04 to 2,031.8) in 2040. Comparatively, in the African region, the incidence rates are expected to reach 184.08 (182.34 to 185.82) in 2020, and 195.94 (177.12 to 215.49) in 2030 per 100,000 population.

Mortality

In Table 2 we describe our findings with respect to the age-standardized, sex-averaged mortality rates per 100,000 with 95% UIs due to AEMT between the years 1990 and 2017 within each WHO region and its respective countries. In 2017, there were 1.60 (1.33 to 1.90) deaths per 100,000 population. This translates to approximately 1.20 million deaths worldwide due to AEMT although reassuringly, death due to AEMT has declined globally from 2.14 (1.68 to 2.39) to 1.60 (1.33 to 1.90) per 100,000 individuals. The global percentage change in mortality rate between 1990 and 2017 due to AEMT was -25.23% (-10.53 to 39.92). The death rates due to AEMT in Ghana [5.07 (3.72 to 6.27)], India [3.66 (2.47 to 4.37)], and Brazil [1.55 (1.43 to 2.01)] were higher compared to Australia [0.99 (0.69 to 1.17)], the United States [0.96 (0.89 to 1.28)], and Sweden [0.94 (0.58 to 1.07)].

**Table 2:** Global Age-standardized mortality rates per 100,000 population due to adverse effect of medical treatment by World Health Organization regions

	Mortality (95% Uncertainty Intervals)				Percentage Change (95% Uncertainty Intervals)		
	1990	2000	2010	2017	1990 to 2017	2000 to 2017	2010 to 2017
Global	2.14 (1.68 - 2.39)	1.91 (1.53 - 2.14)	1.67 (1.38 - 1.94)	1.6 (1.33 - 1.90)	-25.23 (-39.92 to -10.53)	-16.23 (-27.96 to -4.49)	-4.19 (-7.56 to -0.81)
African Region	3.22 (2.53 - 4.22)	3.21 (2.51 - 4.00)	2.73 (2.19 - 3.50)	2.58 (2.09 - 3.33)	-19.87 (-33.15 to -6.58)	-19.62 (-30.61 to -8.62)	-5.49 (-11.98 to 1)
Angola	3.44 (2.38 - 5.00)	3.23 (2.35 - 4.66)	2.66 (1.85 - 3.86)	2.25 (1.5 - 3.36)	-34.59 (-47.87 to -21.3)	-30.34 (-41.31 to -19.36)	-15.41 (-21.91 to -8.9)
Ghana	4.95 (3.75 - 6.58)	4.62 (3.59 - 6.08)	5.09 (3.75 - 6.19)	5.07 (3.72 - 6.27)	2.42 (-11.14 to 15.98)	9.74 (-1.30 to 20.78)	-0.39 (-6.79 to 6.01)
Kenya	2.29 (1.60 - 3.25)	2.39 (1.62 - 3.56)	2.03 (1.45 - 3.08)	1.91 (1.31 - 2.91)	-16.59 (-30.55 to -2.62)	-20.08 (-31.26 to -8.89)	-5.91 (-12.36 to 0.54)
South Africa	1.82 (1.36 - 2.10)	2.27 (1.68 - 2.67)	1.65 (1.39 - 2.08)	1.36 (1.1 - 1.76)	-25.27 (-39.25 to -11.28)	-40.08 (-51.24 to -28.91)	-17.57 (-24.02 to -11.11)
Eastern Mediterranean Region	3.07 (2.12 - 3.74)	2.97 (2.14 - 3.50)	2.68 (2.02 - 3.17)	2.46 (1.85 - 2.96)	-19.86 (-34.09 to -5.62)	-17.17 (-28.21 to -6.12)	-8.2 (-14.62 to -1.77)
Egypt	1.67 (1.05 - 2.10)	1.30(0.83 - 1.69)	1.15 (0.71 - 1.59)	1.08 (0.65 - 1.54)	-35.32 (-49.84 to -20.79)	-16.92 (-28.17 to -5.66)	-6.08 (-12.62 to 0.46)
Saudi Arabia	3.92 (2.69 - 5.36)	3.85 (2.77 - 4.73)	3.98 (2.88 - 5.04)	3.37 (2.23 - 4.66)	-14.03 (-28.72 to 0.66)	-12.46 (-23.94 to -0.97)	-15.32 (-21.99 to -8.64)
European Region	1.53 (1.23 - 1.61)	1.31 (1.11 - 1.48)	1.16 (1.05 - 1.42)	1.1 (1.01 - 1.38)	-28.1 (-43.12 to -13.07)	-16.03 (-27.77 to -4.28)	-5.17 (-11.86 to 1.52)
Germany	1.05 (0.97 - 1.32)	0.83 (0.76 - 1.15)	1.10 (0.89 - 1.24)	1.19 (0.87 - 1.41)	13.33 (-1.98 to 28.64)	43.37 (31.36 to 55.37)	8.18 (1.33 to 15.02)
Kazakhstan	1.41 (1.07 - 1.64)	1.43 (1.08 - 1.60)	1.18 (1.03 - 1.54)	1.22 (1.03 - 1.59)	-13.47 (-28.83 to 1.89)	-14.68 (-25.32 to -4.03)	3.38 (-3.53 to 10.29)
Poland	1.09 (0.91 - 1.33)	1.27 (0.98 - 1.41)	1.26 (0.92 - 1.40)	1.07 (0.86 - 1.31)	-1.83 (-17.59 to 13.93)	-15.74 (-26.66 to -4.81)	-15.07 (-22.12 to -8.01)
Russian Federation	0.89 (0.81 - 1.32)	1.39 (1.04 - 1.46)	1.34 (1.04 - 1.53)	1.13 (0.87 - 1.32)	26.96 (10.85 to 43.06)	-18.7 (-29.91 to -7.48)	-15.67 (-22.77 to -8.56)
Sweden	0.78 (0.67 - 0.93)	0.99 (0.66 - 1.09)	1.07 (0.63 - 1.18)	0.94 (0.58 - 1.07)	20.51 (4.84 to 36.17)	-5.05 (-16.57 to 6.47)	-12.14 (-19.26 to -5.01)
Ukraine	0.58 (0.44 - 0.67)	0.59 (0.44 - 0.67)	0.57 (0.49 - 0.73)	0.85 (0.73 - 1.27)	46.55 (31.21 to 61.88)	44.06 (32.22 to 55.89)	49.12 (41.89 to 56.34)
United Kingdom	0.86 (0.82 - 1.09)	0.99 (0.84 - 1.10)	0.93 (0.79 - 1.05)	1.04 (0.80 - 1.09)	20.93 (7.88 to 33.97)	5.05 (-4.52 to 14.62)	11.82 (3.88 to 19.75)
Region of the Americas	2.10 (1.69 - 2.20)	1.62 (1.47 - 1.91)	1.37 (1.29 - 1.77)	1.33 (1.25 - 1.76)	-36.66 (-48.18 to -25.13)	-17.9 (-26.49 to -9.3)	-2.91 (-10.43 to 4.61)
Argentina	5.34 (3.58 - 5.78)	3.25 (2.99 - 4.37)	3.48 (3.15 - 4.42)	3.27 (2.74 - 4.45)	-38.76 (-50.64 to -26.87)	0.61 (-8.82 to 10.04)	-6.03 (-13.97 to 1.91)
Brazil	2.27 (1.75 - 2.42)	2.04 (1.62 - 2.19)	1.53 (1.41 - 1.95)	1.55 (1.43 - 2.01)	-31.71 (-43.96 to -19.45)	-24.01 (-33.41 to -14.6)	1.3 (-6.5 to 9.10)
Cuba	3.12 (1.5 - 3.49)	1.67 (1.39 - 1.95)	0.77 (0.64 - 1.55)	0.79 (0.62 - 1.49)	-74.67 (-87.41 to -61.92)	-52.69 (-62.46 to -42.91)	2.59 (-5.21 to 10.39)
Mexico	2.14 (1.89 - 2.33)	1.34 (1.24 - 1.96)	1.44 (1.36 - 1.89)	1.39 (1.30 - 1.83)	-35.04 (-46.16 to -23.91)	3.73 (-5.06 to 12.52)	-3.47 (-11.26 to 4.32)
United States	1.27 (1.09 - 1.44)	1.18 (1.06 - 1.41)	0.97 (0.92 - 1.29)	0.96 (0.89 - 1.28)	-24.4 (-36.3 to -12.49)	-18.64 (-27.69 to -9.58)	-1.03 (-8.84 to 6.78)
Southeast Asian Region	3.50 (2.46 - 4.08)	3.36 (2.36 - 3.83)	3.09 (2.2 - 3.47)	2.93 (2.07 - 3.40)	-16.28 (-29.84 to -2.71)	-12.79 (-24.55 to -1.02)	-5.17 (-12.76 to 2.42)
China	1.23 (0.82 - 1.44)	0.94 (0.67 - 1.06)	0.51 (0.44 - 0.70)	0.41 (0.36 - 0.58)	-66.66 (-79.27 to -54.04)	-56.38 (-65.98 to -46.77)	-19.6 (-27.39 to -11.8)
India	4.04 (2.70 - 4.83)	3.94 (2.7 - 4.52)	3.77 (2.59 - 4.28)	3.66 (2.47 - 4.37)	-9.4 (-22.68 to 3.88)	-7.1 (-19.01 to 4.81)	-2.91 (-10.57 to 4.75)
Indonesia	1.92 (1.45 - 2.41)	1.93 (1.37 - 2.46)	1.72 (1.27 - 2.17)	1.56 (1.16 - 2.00)	-18.75 (-32.03 to -5.46)	-19.17 (-31.07 to -7.26)	-9.3 (-16.96 to -1.63)
Western Pacific Region	1.22 (0.86 - 1.39)	0.95 (0.74 - 1.06)	0.65 (0.58 - 0.82)	0.57 (0.52 - 0.72)	-53.27 (-67.79 to -38.74)	-40 (-51.32 to -28.67)	-12.3 (-19.52 to -5.07)
Australia	1.09 (0.82 - 1.19)	1.15 (0.83 - 1.25)	1.05 (0.76 - 1.15)	0.99 (0.69 - 1.17)	-9.17 (-23.13 to 4.79)	-13.91 (-25.44 to -2.37)	-5.71 (-13.29 to 1.87)
Fiji	2.43 (1.83 - 3.02)	2.51 (1.97 - 3.00)	2.34 (1.88 - 2.80)	2.17 (1.68 - 2.73)	-10.69 (-24.67 to 3.29)	-13.54 (-25.05 to -2.02)	-7.26 (-14.68 to 0.16)
Japan	0.42 (0.39 - 0.54)	0.45 (0.39 - 0.54)	0.53 (0.38 - 0.57)	0.50 (0.36 - 0.56)	19.04 (4.80 to 33.27)	11.11 (0.00 to 22.22)	-5.66 (-13 to 1.68)

**Figure 4:**
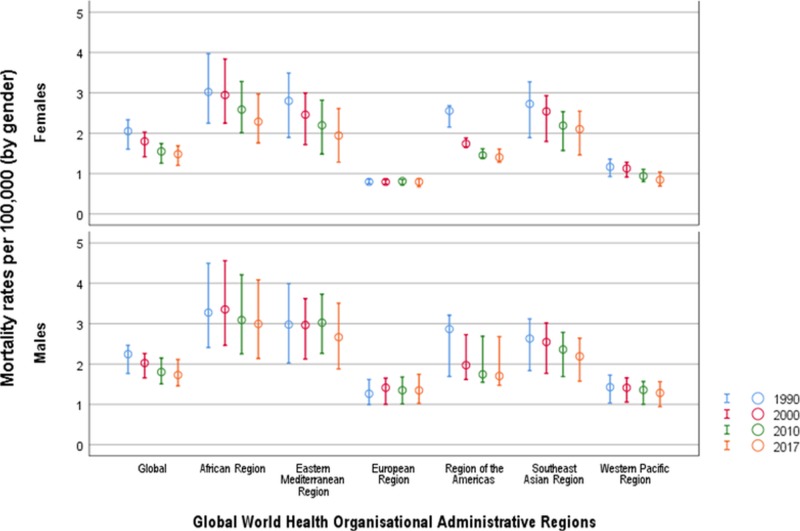
Age-standardized mortality rates due to adverse effect of medical treatment by Global World Health Organization regions between 1990 and 2017 Mortality rates are per 100,000 population. Circles represent summary estimates of mortality and error bars represent 95% uncertainty intervals.

In Figure 4 we show a general decline in mortality rates caused by AEMT by WHO regions between 1990 and 2017. The greatest decline in the percentage change of mortality rates across the WHO regions was noted in the Western Pacific region [-53.27% (-67.79 to -38.74)], compared with the smallest change in the Southeast Asian Region [-16.28 (-29.84 to -2.71)]. The annualized rate of mortality change between 1990 and 2017 was -0.90% and global trends in mortality due to AEMT are decreasing exponentially, with the percentage of annualizing mortality change between 2010 and 2017 standing at approximately -0.52%. There was a decline in mortality rates during 1990 to 2017 in Cuba [-74.67% (-87.41 to -61.92)], China [-66.66% (-79.27 to -54.04)], Egypt [-35.32% (-49.84 to -20.79)], Mexico [-35.04% (-46.16 to -23.91)], and Angola [-30.34% (-41.31 to -19.36)]. However, in contrast, there was an increase in the percentage change between 1990 and 2017 in mortality rates in countries such as Ukraine [46.55% (31.21 to 61.88)], the United Kingdom [20.93% (7.88 to 33.97)], Sweden [20.51% (4.84 to 36.17)], and Japan [19.04% (4.8 to 33.27)].

When we compare countries within the WHO regions between 2010 and 2017, increasing trends in mortality rates were seen in Ukraine [49.12 (41.89 to 56.34)], the United Kingdom [11.82% (3.88 to 19.75)], and Germany [8.18% (1.33 to 15.02)]. Decreasing mortality was noted in China [-19.6% (-27.39 to -11.8)], South Africa [-17.57% (-24.02 to -11.11)], the Russian Federation [-15.67% (-22.77 to -8.56)], and Angola [-15.41% (-21.91 to -8.90)].

Mortality by age and gender

**Figure 5:**
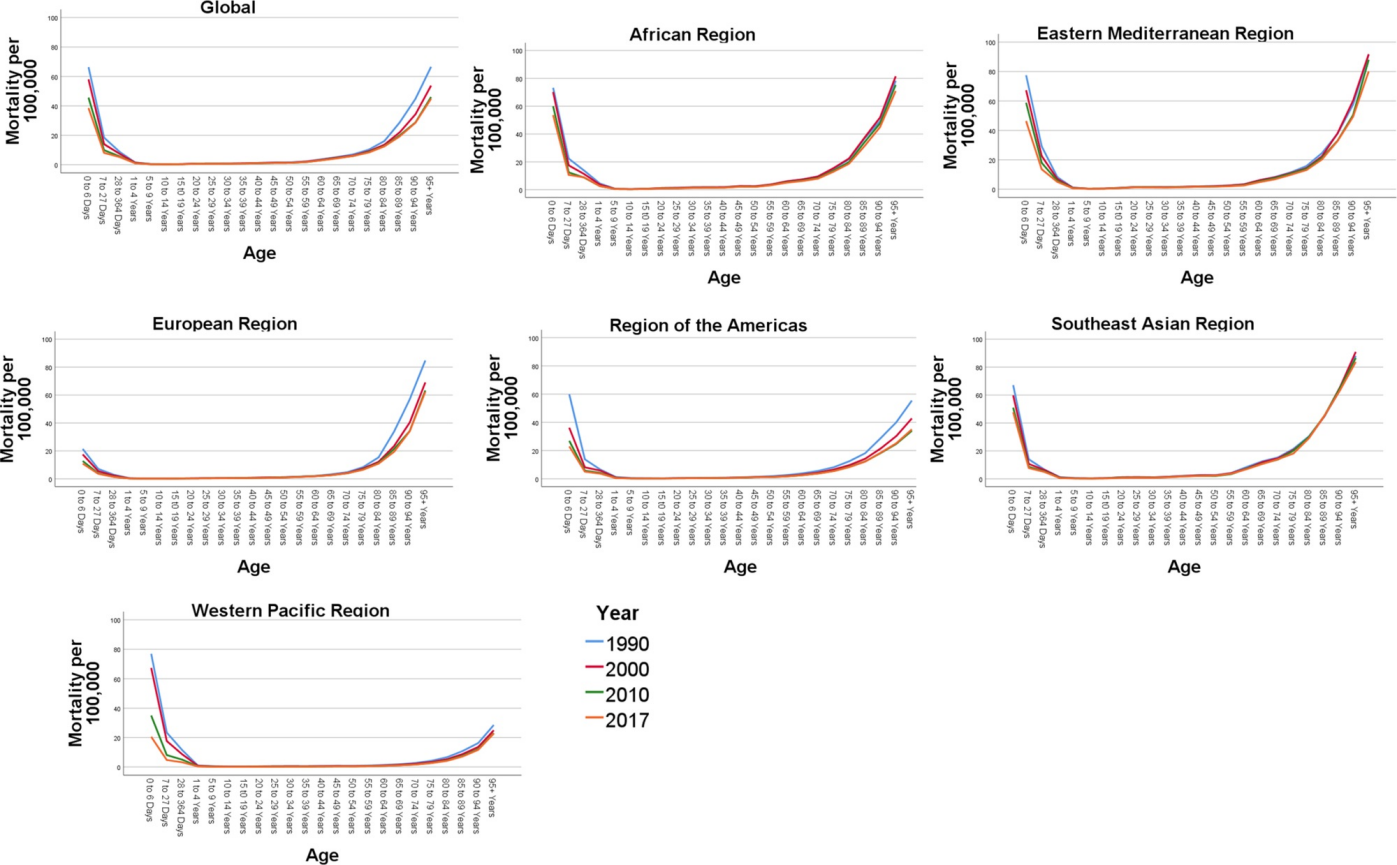
Age-specific mortality rates due to adverse effect of medical treatment by Global World Health Organization regions between 1990 and 2017 The figure panes depict the overall mortality rates per 100,000 population globally and across the six World Health Organization (WHO) regions (African region, Eastern Mediterranean region, European Region, Region of the Americas, Southeast Asian Region and the Western Pacific Region) during the year 1990, 2000, 2010 and 2017.

In Figure 5 we illustrate trends in age-specific mortality rates from AEMT between 1990 and 2017 by WHO region. The age-specific mortality rate shows a bimodal increase between the age group of zero to one year, and an increase from the age group of 65 years and over. The highest age-specific mortality rate globally was observed in the age group of 95 years and over, with a mortality rate of 42 per 100,000. However, in the Southeast Asian region, increased mortality rates were observed starting from the age group of 65 years and over. Globally, we observed the highest mortality rate due to AEMT among newborns between zero and six days old.

Overall, the AEMT mortality rate was higher in men (1.73 per 100,000) compared to women (1.48 per 100,000). In the Southeast Asian region, women had a slightly higher rate of deaths (2.99 per 100,000) compared to men (2.89 per 100,000). In addition, there was an increased death rate in women in the Eastern Mediterranean region of 2.53, compared to 2.39 per 100,000 in men.

Forecasted mortality trend

**Figure 6:**
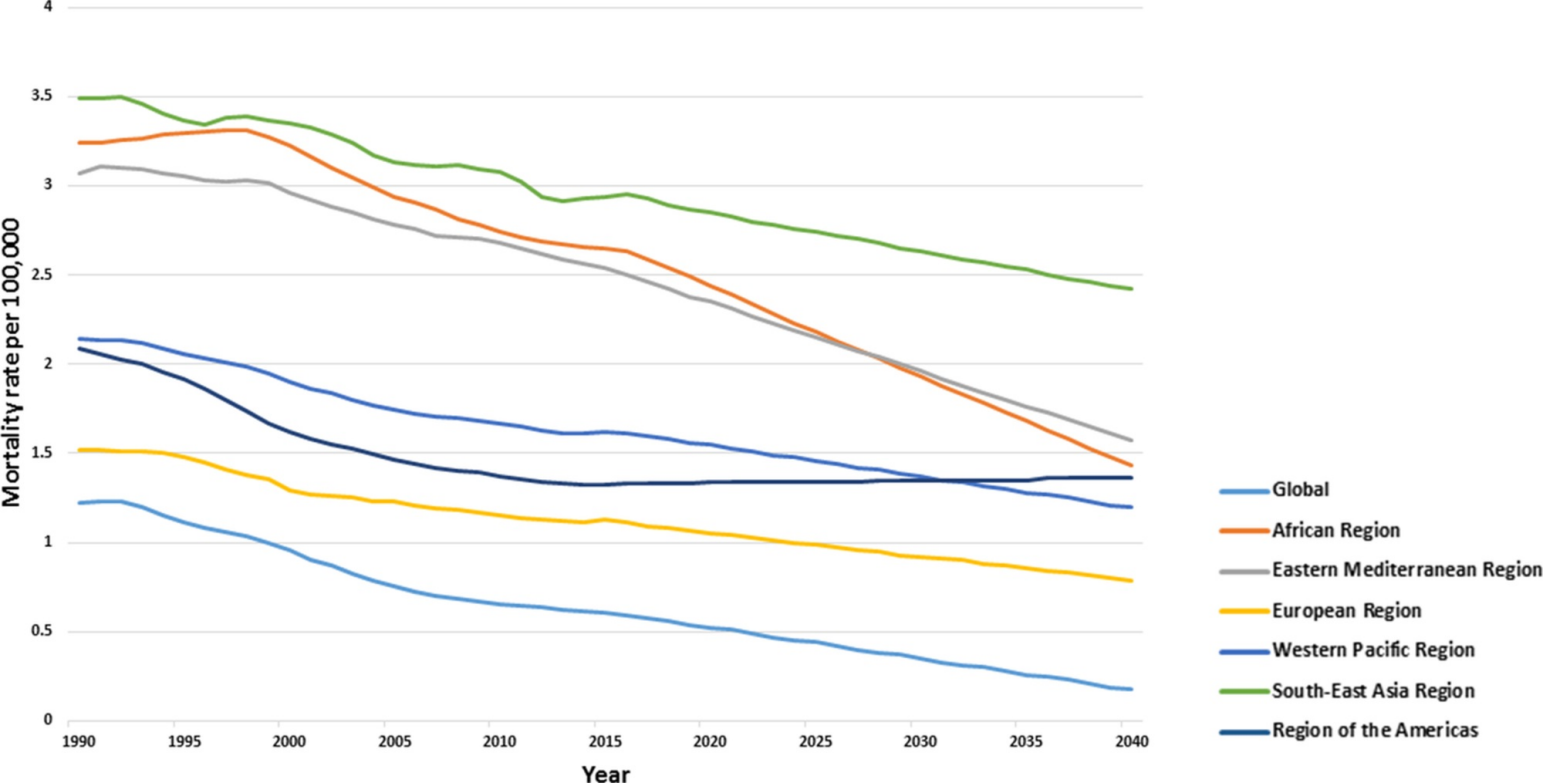
Forecasted age-standardized mortality trends across Global World Health Organization regions till 2040

Although fatal AEMTs were forecasted up until 2040, and a stable declining trend is expected across all WHO regions (Figure 6). The global mortality rate due to AEMT is predicted to be 1.55 (1.48 to 1.61) in 2020, 1.37 (0.88 to 1.86) in 2030 and 1.2 (0.08 to 2.32) deaths per 100,000 by 2040. Across the WHO regions, the highest predicted mortality rates are observed in Southeast Asia, with deaths due to AEMT by 2020 expected to be 2.85 (2.72 to 2.97), while the lowest predicted mortality is seen in the Western Pacific region [0.52 (0.45 to 0.60)]. In 2030, higher mortality rates are forecasted in Southeast Asia [2.63 (2.34 to 2.92)], followed by the Eastern Mediterranean [1.96 (1.12 to 2.8)]. By 2040, the mortality rates are expected to decline in the Southeast Asian region [2.42 (2.03 to 2.81)], the Eastern Mediterranean region [1.57 (-0.36 to 3.5)], the region of the Americas [1.36 (0.13 to 2.60)], the African region [1.43 (-0.40 to 3.26)], and the European region [0.79 (-0.22 to 1.80)].

## Discussion

In the current study, we aimed to describe trends, levels, and patterns of incidence and mortality due to AEMT by WHO administrative regions and the selected countries between 1990 and 2017. This study has revealed that although the incidence of AEMT increased between 1990 and 2017, the AEMT burden due to mortality has significantly improved. One of the core findings of our study is the increased incidence in both developed regions and developed countries, and specifically increased mortality due to AEMT in lower-income and developing WHO regions. We observed a higher incidence in women and higher mortality rates in men due to AEMT. In addition, there is a higher occurrence of AEMT events for children under one year old, and for elderly over 65 years of age.

The findings of our study using the GBD data suggest an estimated 40% increase over the last three decades in the incidence of adverse effects of medical care. We also observed a wide variation in incidence rates across different regions in 2017. Only 181 new cases per 100,000 due to AEMT were reported in the African region, compared to over 1,160 per 100,000 reported in the Americas. However, earlier reports suggest that these numbers may be underestimated because of non-reporting of new cases of AEMT [21]. In addition, the forecasted incidence in the African region is the lowest across all regions globally for 2020, 2030, and 2040. The results of our study are in line with previous reports, where under-reporting and the wide variation of incidence reporting of AEMT is prevalent in both developing and developed countries [6,11]. The lesser accountability of AEMT incidence could be due to regional factors affecting healthcare systems, attitudes of the providers, methodological issues, and a lack of the required resources to document these events across some global regions. The higher incidence rates in extreme ages (<one year and 65 years and over) identified in our study are alarming. Inappropriate prescribing in the elderly including errors in the dosage of medication per weight and the related side effects of medications in neonates are the most likely explanations for such a high incidence rates in these age groups [9,22].

Our study has also demonstrated that reducing the number of new occurrences of AEMT is challenging, even in high-income countries such as the United States and Australia. Nevertheless, declining mortality rates between 1990 and 2017 indicate improved clinical care. Despite a 25% reduction in global mortality due to AEMT between 1990 and 2017, mortality varied widely across regions, with significant reductions in the Western Pacific region compared to the Southeast Asian region. The countries in the Southeast Asia region, such as India and China, are undergoing accelerated socio-economic changes, with rapid transition of healthcare systems resulting in significant challenges to addressing health needs [23]. Although China has managed to reduce death rates by 67%, India still has a long way to go, as the death rate was reduced by only 9% between 1990 and 2017. Interestingly, between 1990 and 2007, Cuba achieved a 75% reduction in mortality rates due to AEMT. These reductions in fatal AEMT are encouraging and are consistent with the policies of the National Healthcare System, which values improvement through continuous assessment and evaluation of risks to reduce AEMT [24]. Such national policies should be further presented in global health forums for the provision of universal, equitable systems to ensure excellent care and a reduction in health inequalities. Such system-wide action to provide an equitable National Healthcare system is needed when there are epidemiological transitions due to different growth and socio-economic trends across the WHO regions.

Surprisingly, the United Kingdom has a positive trend in the mortality rate of 20.93% between 1990 and 2017. However, the result should be viewed with caution because the death rates per 100,000 population in the United Kingdom were 0.86 (0.82 to 1.09) in 1990, and 1.04 (0.8 to 1.09) in 2017. These numbers are low for the European region average: 1.1 (1.01 to 1.38) deaths/100,000. In addition, the number of fatal AEMTs in the United Kingdom was lower than in similarly developed countries such as Germany [1.19 (0.87 - 1.41)]. A possible explanation for the high mortality due to AEMT could be an increasing burden of the aging population in these countries.

The forecasted rising incidence of AEMT across all WHO regions has several important implications for health policymakers, healthcare professionals and the public. Despite significant progress in healthcare in the last 30 years, there has been an increased incidence of AEMT, which calls for fresh policies to be implemented at the institutional, national, and WHO regional levels [10].

Human error is inevitable while providing medical care. Nevertheless, adverse events may be preventable and avoidable in up to 80% whenever standard care is observed by physicians [6]. Nevertheless, evolving healthcare needs and newer healthcare goals due to the advancement of health research have put healthcare professionals on a trajectory of increased AEMT. Therefore, high-quality healthcare systems that can contextualize and respond to the needs of the population efficiently are required and strategies should be employed to reduce negligence or substandard care [9].

In today’s world, the value of the use of electronic medical records (EMR) cannot be underestimated, even in developing countries where such tools can be easily accessible and it is feasible to incorporate them in the provision of clinical healthcare [25]. Such tools are likely to enhance medical care by providing an accurate list of medications and their side effects and by incorporating clinical decision-making tools that may help to improve healthcare standards. Moreover, innovative methods such as artificial intelligence (AI), machine learning, and deep learning neural networks have been ingrained into the healthcare system. The current trends of AI, advancing computing power, and machine learning algorithms are expected to play an essential role in reducing AEMT due to the availability of big data [26]. Such new concepts and tools should be gradually incorporated into medical care to prevent AEMT in complicated clinical settings in developed regions.

Our study has some limitations. We used secondary data obtained from the GBD study. Although the methodology of GBD study is meticulous with a clear framework, there is a possibility of non-comparable information in the GBD 2017 estimates due to non-coding or under-reporting of adverse events, especially in developing countries and under-resourced WHO regions. In addition, changes in the case definition and heterogeneity in study designs could influence our results. Moreover, ICD codes 9 and 10 differ for AEMT, and the GBD study could have underestimated or overestimated the burden of AEMT. However, the GBD 2017 study used robust methodological techniques to provide reliable estimates for the outcomes, picturing a holistic view of the AEMT epidemiology across the WHO regions and countries rather than obtaining data from a few clinical settings [27]. We presented the results for selected countries according to the WHO geographical regions. The administrative regions have representatives from each country within the region. The delegates from member states will meet to discuss health issues pertaining to the geographic region, develop technical cooperation strategies for regional health development, and provide policies for health promotion and disease and injury prevention. Nevertheless, future studies with a detailed account of the AEMT burden in individual countries within the WHO regions are warranted.

## Conclusions

AEMT continues to increase in occurrence, leading to increased morbidity. Little progress has been achieved to curtail the incidence rates of AEMT. Although the number of fatal AEMTs has been reduced globally over the last 30 years, there is a wide variation among different geographical regions. The high burden of AEMT warrants the implementation of robust policies, re-design of service deliveries, transformation of the healthcare workforce through appropriate patient safety training, improvement of accountability for quality and patient safety by clearer and transparent documentation, and development of a safe culture of feedback using EMRs to achieve the WHO’s patient safety strategy goals.
